# A Synergistic Combination of Niclosamide and Doxorubicin as an Efficacious Therapy for All Clinical Subtypes of Breast Cancer

**DOI:** 10.3390/cancers13133299

**Published:** 2021-06-30

**Authors:** Garima Lohiya, Dhirendra S. Katti

**Affiliations:** 1 Department of Biological Sciences and Bioengineering, Indian Institute of Technology Kanpur, Kanpur 208016, Uttar Pradesh, India; glohiya@iitk.ac.in; 2 Mehta Family Centre for Engineering in Medicine, Indian Institute of Technology Kanpur, Kanpur 208016, Uttar Pradesh, India

**Keywords:** Niclosamide, doxorubicin, breast cancer, Wnt/β-catenin signaling, combination therapy, cell cycle arrest

## Abstract

**Simple Summary:**

Chemotherapy is the gold standard treatment option for metastatic cancers. However, the efficacy of chemotherapy is limited due to the development of resistance. The aberrantly expressed Wnt/β-catenin signaling pathway acts as one of the major cancer drivers that also causes the development of resistance. Therefore, in this study, we explored the combinatorial approach of downregulating the Wnt/β-catenin pathway along with using a chemotherapeutic agent as a strategy to overcome drug resistance and improve cancer therapy. We evaluated the combinatorial efficacy of Niclosamide (an antihelminthic repurposed as a Wnt signaling inhibitor) and Doxorubicin (first-line treatment for multiple cancers in the clinic) against breast cancer. The combination showed synergistically enhanced death of all three clinical subtypes of breast cancer cells in both the sequential and concurrent treatment regimens and holds the potential to be developed as an efficient therapeutic option for breast cancer irrespective of its clinical subtype.

**Abstract:**

Drug resistance is one of the major hurdles in the success of cancer chemotherapy. Notably, aberrantly expressed Wnt/β-catenin signaling plays a major role in the initiation and maintenance of oncogenesis along with development of chemoresistance. Therefore, the combinatorial approach of targeting Wnt/β-catenin pathway along with using a chemotherapeutic agent seems to be a promising strategy to improve cancer therapy. In the present study, we evaluated the combination of niclosamide (Nic), an FDA-approved antihelminthic drug repurposed as a Wnt signaling inhibitor, and doxorubicin (Dox), a conventional anticancer agent, in all clinical subtypes of breast cancer viz. triple negative breast cancer, HER2 positive breast cancer, and hormone receptor positive breast cancer. The results demonstrated that the combination induced apoptosis and caused synergistically enhanced death of all breast cancer cell types at multiple combinatorial concentrations using both the sequential and concurrent treatment regimens. Mechanistically, downregulation of Wnt/β-catenin signaling and cell cycle arrest at G0/G1 phase by Nic and increase in reactive oxygen species by both Nic and Dox along with the inherent cytotoxicity of Dox mediated the synergism between the two drugs in both the treatment regimens. Overall, the combination of Nic and Dox holds promise to be developed as an efficient therapeutic option for breast cancer irrespective of its clinical subtype.

## 1. Introduction

Chemotherapy remains the main therapeutic option for treating metastatic cancers in the clinic. However, drug resistance is one of the major hurdles in the success of cancer chemotherapy [1]. The reasons for the development of resistance are multifactorial, which include alterations in the biological and biochemical characteristics of cancer cells [2]. Few examples of such alterations include increased drug efflux by ATP-dependent pumps, enhanced DNA repair, inactivation of apoptotic pathways, altered intracellular drug targets, and mutations of cell surface targets [3]. It is noteworthy that different mechanisms that regulate drug resistance are governed by distinct cell signaling pathways [4]. Therefore, drug resistance is a gene-driven and signaling pathway-mediated process. Multiple signaling pathways such as PI3K/AKT/mTOR, Notch, Hedgehog, NF-κB, TFG-β, Ras/MAPK, JAK/STAT, and Wnt have been reported to be dysregulated in cancer [4]. Along with drug resistance, these dysregulated signaling pathways are also known to be the underlying cause of initiation, progression, and maintenance of oncogenesis [5]. Therefore, targeting dysregulated signaling pathways will not only help in overcoming drug resistance in cancer cells, but will also make them less oncogenic and chemo-sensitive. When treated with anti-cancer drugs, such chemo-sensitive cells with decreased oncogenic potential are expected to undergo increased apoptosis and enable a better therapeutic outcome [6,7]. Hence, a combinatorial approach of targeting dysregulated signaling pathways along with using chemotherapeutic drugs may be an efficient approach to improve the therapeutic outcome for cancer patients.

Wnt/β-catenin signaling is one of the aberrantly expressed signaling pathways in multiple cancers, which is known to be associated with development of resistance against treatment [8,9]. Moreover, aberrant Wnt signaling initiates oncogenesis by maintaining cancer stem cells (CSCs) and causes immune evasion and epithelial to mesenchymal transition (EMT), all of which are implicated in disease progression and failure of the treatment methods [10]. Therefore, Wnt signaling is a potential therapeutic target to overcome drug resistance and to improve cancer treatment [11]. Recent studies demonstrate that Niclosamide (Nic), an FDA-approved antihelminthic drug, has shown promising results as a Wnt signaling inhibitor and an anticancer agent [12,13]. Nic is capable of efficiently downregulating Wnt signaling in multiple cancer cells such as breast cancer, prostate cancer, colorectal cancer, lung cancer, and ovarian cancer [12,13,14]. Further, being an FDA-approved antihelminthic drug, its safety and pharmacokinetic profile for this specific application have previously been established. Therefore, repurposing Nic for cancer therapy is translationally less challenging, making it a good choice to target Wnt signaling.

In the present study, we aimed to develop a combination therapy based on Nic and a conventional anti-cancer agent to provide therapeutic benefits related to drug resistance and disease recurrence. We hypothesized that inhibiting aberrantly activated Wnt signaling by Nic would impart chemosensitivity to cancer cells thereby increasing the efficacy of conventional anti-cancer drugs. As a combination partner for Nic, Doxorubicin (Dox), an anthracycline that is used as a first-line treatment drug for multiple cancers in the clinic, was chosen.

Breast cancer is the most common and prevalent cancer across the globe. Moreover, breast cancer is the leading cause of cancer-related deaths amongst women worldwide. This emphasizes the need for the better management of the disease. With this motivation, in the present study, among various types of cancers harboring aberrantly activated Wnt signaling, breast cancer was chosen to test the combinatorial efficacy of Nic and Dox. Moreover, the combination was tested on all the three clinical subtypes of breast cancer viz. triple negative breast cancer (TNBC), HER2 positive breast cancer, and hormone receptor (HR) positive breast cancer, as a common targeted therapy effective in all the subtypes of breast cancer is not yet available. Further, keeping in mind the heterogeneity of breast cancers as well as clinical requirement of cancer patients, two regimens of combination therapy, sequential therapy (Nic → Dox) and concurrent therapy (Nic + Dox), were studied [15]. Since in sequential therapy one drug is administered at a time, it may show better patient compliance for elderly patients who have reduced ability to tolerate the toxicity of multiple drugs at a time or for patients with co-morbidities or in patients with slow growing tumors [15,16], whereas concurrent therapy will be useful for younger patients or for patients who require urgent reduction in their tumor burden or display fast tumor growth [16]. Therefore, the developed combinatorial therapy was evaluated in both regimens to meet the needs of a diverse set of patients.

The results demonstrated that the individual treatment of Nic and Dox caused significant death of all breast cancer cells. However, combination of Nic and Dox was more potent in inducing apoptosis and caused synergistically enhanced death of all clinical subtypes of breast cancer cells at multiple combinatorial concentrations in both the treatment regimens (sequential and concurrent). Moreover, elucidation of the mechanism suggested that the enhanced efficacy of the combination was mediated by downregulation of Wnt/β-catenin signaling, cell cycle arrest at the G0/G1 phase, and increased ROS generation in all breast cancer cells.

## 2. Materials and Methods

### 2.1. Cell Culture

MDA-MB-231 and SKBR3 cells were maintained in DMEM/F12 (Gibco; Thermo Fisher Scientific, Inc., Waltham, MA, USA) supplemented with 10% FBS (Gibco; Thermo Fisher Scientific, Inc., Waltham, MA, USA), 0.1% penicillin-streptomycin (Hi-Media, Mumbai, Maharashtra, India), 0.1% amphotericin (Hi-Media, Mumbai, Maharashtra, India), and 0.1% ciprofloxacin (Ranbaxy Laboratories Ltd., Gurugram, Haryana, India) in humidified incubator with 5% CO_2_ at 37 °C. MCF7 cells were maintained in DMEM (Gibco; Thermo Fisher Scientific, Inc., Waltham, MA, USA) supplemented with 10% FBS (Gibco; Thermo Fisher Scientific, Inc., Waltham, MA, USA) and 1% penicillin-streptomycin (Hi-Media, Mumbai, Maharashtra, India) in a humidified incubator with 5% CO_2_ at 37 °C. Cell lines used in the study were tested for absence of Mycoplasma contamination and validated by morphological observation. All the experiments were performed post 24 h of cell seeding unless otherwise stated.

### 2.2. Cytotoxicity Analysis of Niclosamide (Nic) and Doxorubicin (Dox) Individually

To assess the cytotoxicity of Nic and Dox alone, breast cancer cell lines MDA-MB-231, SKBR3, and MCF7 seeded in 96-well cell culture plate at a density of 5000 cells/well, were treated with different concentrations of Nic and Dox individually for 24 h and 48 h in complete cell culture medium. Dox stock was made in saline and Nic stock was made in DMF. Appropriate vehicle controls were kept in the case of Nic, and the maximum vehicle concentration used was 0.1%. Post-treatment, cell viability was assessed using resazurin assay. In this, cells were incubated with resazurin reagent (Sigma Aldrich, St. Louis, MO, USA) in complete media at a concentration of 0.02 mg/mL for 5 h and then fluorescence was measured at an excitation of 540 nm and emission of 600 nm using a multi-mode plate reader (Synergy H4, BioTek Instruments, Inc., Winooski, VT, USA). The data were analyzed as percentage viability with respect to the untreated control. IC_50_ values were calculated through non-linear regression analysis in Prism (version 6.01, GraphPad Software).

### 2.3. Cytotoxicity Analysis of the Combination of Niclosamide (Nic) and Doxorubicin (Dox)

To assess the cytotoxicity of the combination of Nic and Dox, a similar protocol as discussed above for individual drug cytotoxicity analysis was used. Briefly, 5000 cells/well seeded in a 96-well cell culture plate were treated with different concentrations of Nic and Dox in sequential (Nic for 24 h followed by Dox for another 24 h) and concurrent (Nic and Dox together for 48 h) treatment regimens, and cytotoxicity of the combination was evaluated using resazurin assay. To calculate the combination index of various combinatorial concentrations, firstly, inhibitory concentrations of individual drugs (IC_F_) were calculated using the following equation (known as IC_anything_; sourced from non-linear regression analysis of dose–response curve in Prism, Graph Pad software)

(1) $${\mathrm{IC}}_{F\;}={\left(\frac{F}{100-F}\right)}^{1/H}\times {\;\mathrm{IC}}_{50}\;$$

where:

IC_F_ = inhibitory concentration of individual drug (Nic or Dox) required to cause the same percentage of cell death as caused by the combination of drugs (Nic and Dox).
F = percentage of viable cells left after treatment with the combination (sequential or concurrent) of Nic and Dox.
100-F = percentage of dead cells after treatment with the combination (sequential or concurrent) of Nic and Dox.
H = hill slope of non-linear regression of individual drug cytotoxicity curve.
IC_50_ = inhibitory concentration of individual drug required to cause 50% cell death.

After calculating the IC_F_ values for both Nic (IC_FNic_) and Dox (IC_FDox_), CI for various combinations of Nic and Dox was calculated using the following formula [17]

(2) $$\mathrm{CI}\left(F\right)=\frac{\mathrm{Nic}\left(\mathrm{Fcombo}\right)}{{\mathrm{IC}}_{\mathrm{FNic}}\;}+\frac{\mathrm{Dox}\left(\mathrm{Fcombo}\right)}{{\mathrm{IC}}_{\mathrm{FDox}\;}}$$

where:

CI(F) = combination index of a combination of Nic and Dox which caused (100- F) percentage of cell death.
Nic (Fcombo) = concentration of Nic required to cause (100-F) percentage of cell death when used in combination.
Dox (Fcombo) = concentration of Dox required to cause (100-F) percentage of cell death when used in combination.

To improve clarity and highlight trends in the data, cell viability as well as CI data were plotted in two formats (heat maps and line plots). All the analyses were performed using mean values determined from three experimental repeats.

### 2.4. Apoptosis Analysis

Apoptosis analysis was performed in cells treated with selected doses of Nic and Dox using Muse^®^ Annexin V and Dead Cell kit (Merck, Millipore, Burlington, MA, USA) according to the manufacturer’s protocol. Briefly, treated cells were washed with 1X phosphate buffer saline (PBS), trypsinized, and transferred into 1.5 mL micro-centrifuge tubes. One hundred microliters of cell suspension were mixed with 100 µL of Muse^®^ Annexin V and Dead Cell reagent and incubated for 20 min at room temperature (RT) in the dark. Later, cells were analyzed in Guava^®^ Muse^®^ Cell Analyzer (Merck, Millipore, Burlington, MA, USA).

### 2.5. Immunofluorescence Staining

For immunostaining of β-catenin, breast cancer cells seeded on glass coverslips were treated with Nic for 24 h and 48 h. Post treatment, cells were fixed using 4% formaldehyde and blocked for 2 h at RT. Later, cells were incubated with β-catenin primary antibody (Sino Biological, Beijing, China) for 2 h at RT, washed three times with 1X PBS, and then incubated with anti-rabbit secondary antibody for 2 h at RT. At last, cells were stained with nuclear stain DAPI (Sigma Aldrich, St. Louis, MO, USA) for 10 min at RT and mounted with anti-fade agent DABCO (Sigma Aldrich, St. Louis, MO, USA). The cells were then imaged using a confocal microscope (LSM780NLO, Carl Zeiss, GmbH-). Nuclear translocation of β-catenin was quantified using a MATLAB software.

### 2.6. Gene Expression Analysis through RT-PCR

Gene expression analysis of various Wnt signaling markers was performed in all the breast cancer cell lines after treatment with the selected concentrations of Nic either alone or in combination with Dox through real-time qPCR. The total RNA was extracted using TRI reagent^®^ (Sigma Aldrich, St. Louis, MO, USA) according to the manufacturer’s protocol. Briefly, treated cells were trypsinized and lysed with 700 µL TRIZOL followed by the addition of 140 µL of chloroform and centrifugation at 13,000 rpm at 4 °C for 15 min. The aqueous phase obtained after centrifugation was pipetted out into fresh 1.5 mL micro-centrifuge tubes and total RNA was pelleted down by adding an equal volume of isopropanol followed by centrifugation at 13,000 rpm for 15 min at 4 °C. The RNA pellet obtained was washed two times with 75% chilled ethanol and allowed to dry at 37°C to remove excess ethanol. The dried pellet was resuspended in RNase-free water and quantified for total RNA using a Nanodrop (Thermo Scientific, Waltham, MA, USA). Complementary DNA (cDNA) was synthesized from 500 ng of total RNA using a cDNA reverse transcription kit (Applied Biosystems, Waltham, MA, USA) according to the manufacturer’s protocol. Subsequently, quantitative PCR was performed in StepOne Plus RT-PCR (Applied Biosystems, Waltham, MA, USA) using SYBR Green Master Mix. The relative expression of the target gene was calculated using the ΔΔCt method by normalizing the Ct of the target gene to the average Ct of the housekeeping gene GAPDH. The fold change expression of the target gene was calculated as 2 (−ΔΔCt) with respect to untreated controls. The primer sequences used are listed in Table S1.

### 2.7. Cell Cycle Analysis

Cell cycle analysis was performed in all the breast cancer cells after individual and combination (sequential and concurrent) treatment of Nic and Dox using Muse^®^ Cell cycle kit (Merck, Millipore, Burlington, MA, USA) according to the manufacturer’s protocol. Briefly, treated cells were trypsinized, transferred into 1.5 mL micro-centrifuge tubes, and pelleted down. Cell pellets were washed with 1X PBS and resuspended in 50 µL of 1X PBS. Resuspended cells were added dropwise into a tube containing 1 mL of ice-cold 70% ethanol and left for at least 3 h for fixation at −20°C. The ethanol-fixed cells were centrifuged and washed twice in 1X PBS. Finally, cells were resuspended in 200 µL of Muse^®^ cell cycle reagent and incubated for 30 min at RT and thereafter analyzed in Guava^®^ Muse^®^ Cell Analyzer (Merck, Millipore, Burlington, MA, USA).

### 2.8. Reactive Oxygen Species (ROS) Analysis

Nic- and Dox-treated cells were analyzed for ROS using DCFDA (Thermo Scientific, Waltham, MA, USA) and DHE (Abcam, Cambridge, MA, USA) reagents according to the manufacturer’s protocol. In DCFDA assay, cells were incubated with 50 µM of DCFDA reagent in HBSS for 45 min in CO_2_ cell culture incubator at 37 °C. After this, cells were rewashed with HBSS and treated with Nic and Dox for 4 h and 8 h in HBSS containing 10% FBS. Fluorescence was then measured using a multi-mode plate reader (Synergy, Hybrid H4, BioTek Instruments, Inc., Winooski, VT, USA) without removing treatment at excitation of 485 nm and emission of 535 nm.

In DHE assay, seeded cells were treated with Nic and Dox for 4 h and 8 h in HBSS containing 10% FBS. An hour before completion of treatment, 20 µM DHE reagent was added in each well. Later, the treatments along with DHE were replaced with HBSS buffer and the fluorescence signal was immediately measured using a multi-mode plate reader (Synergy, Hybrid H4, BioTek Instruments, Inc., Winooski, VT, USA) at 500 nm excitation and 590 nm emission.

### 2.9. Statistical Analysis

Each experiment was repeated at least three times (biological repeat) with at least three technical repeats. Unless otherwise stated, results have been presented as the mean ± standard error of the mean (S.E.M). Student’s *t*-test (unpaired and two-tailed) was used to compare two groups. One-way ANOVA was used for multiple comparisons (when more than two groups were compared). Two-way ANOVA was used for multiple comparisons (when multiple groups and two parameters were compared). All statistical analyses were carried out with Prism (version 6.01, GraphPad Software). A probability (*p*) value less than 0.05 (*p* < 0.05) was considered statistically significant.

## 3. Results

### 3.1. Effect of Sequential Treatment of Nic and Dox (Nic → Dox) on Breast Cancer Cells

#### 3.1.1. Cytotoxicity Analysis of Individual versus Sequential Combination of Nic and Dox

**Figure 1:**
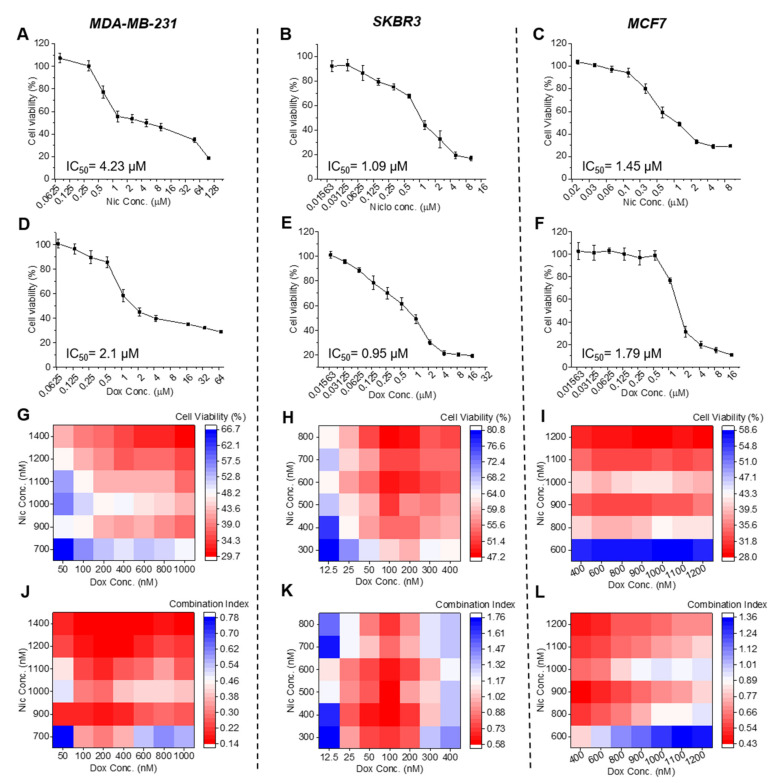
Effect of individual and sequential treatment of niclosamide (Nic) and doxorubicin (Dox) on breast cancer cells. In vitro cytotoxicity of individual (**A**–**C**) Nic and (**D**–**F**) Dox on cells of all three subtypes of breast cancer at different doses for 24 h (*n* = 3). Cell viability heat maps of combination therapy (24 h of Nic treatment followed by 24 h of Dox treatment) in (**G**) MDA-MB-231, (**H**) SKBR3, and (**I**) MCF7 cells. Combination index heat maps in (**J**) MDA-MB-231, (**K**) SKBR3, and (**L**) MCF7 cells.

All three subtypes of breast cancer cells viz. MDA-MB-231 (TNBC), SKBR3 (HER2 positive), and MCF7 (Hormone receptor positive) were treated with different concentrations of Nic (Figure 1A–C) and Dox (Figure 1D–F) individually for 24 h and their in vitro cytotoxicity was assessed. As shown in Figure 1A–F, in- vitro cytotoxicity of individual Nic and Dox demonstrated concentration-dependent death in all subtypes of breast cancer cells with the higher concentrations of drugs causing more cell death. Based on their cytotoxicity data, IC_50_ values were calculated, which suggested that IC_50_ values of Nic were 4.23, 1.09, and 1.45 µM and IC_50_ values of Dox were 2.1, 0.95, and 1.79 µM for MDA-MB-231, SKBR3, and MCF7 cells, respectively.

Later, to examine the effects of sequential combination of Nic and Dox on breast cancer cells, all the three cell lines were treated sequentially with 42 different combinatorial concentrations of Nic and Dox (Figure 1G–I and Figure S1). Concentrations of Nic and Dox used in the combination were decided based on the IC_50_ values of each drug and were lesser than their respective IC_50_ values. As is evident from the cell viability heat maps, cytotoxicity analysis of the sequential combination of Nic and Dox revealed significant death of all the breast cancer cell types at all the combinatorial concentrations studied (Figure 1G–I). The results suggested that most of the pairs of Nic and Dox studied caused more than 50% death in MDA-MB-231 and MCF7 cells and more than 40% death in SKBR3 cells (Figure 1G–I). More specifically, in MDA-MB-231 cells, higher concentrations of Nic (1100–1400 nM) combined with almost all the concentrations of Dox (100–1000 nM) showed relatively higher cytotoxicity (Figure 1G and Figure S1A). In SKBR3 cells, intermediate to higher concentrations of Nic (500–800 nM) combined with intermediate to higher concentrations of Dox (100–400 nM) were more cytotoxic (Figure 1H and Figure S1B), and in MCF7 cells, all the concentrations of Nic (800–1200 nM) except 600 nM combined with all concentrations of Dox (400–1200 nM) showed relatively higher cytotoxicity (Figure 1I and Figure S1C). In order to analyze drug interaction and quantify the efficacy of the combination, combination indices (CI) of each pair of Nic and Dox studied were calculated. It is known that two drugs are synergistic if CI < 1, additive if CI = 1, and antagonistic if CI > 1 [18]. As shown in Figure 1J, it was found that for MDA-MB-231 cells, all 42 combinatorial concentrations of Nic and Dox studied in sequential therapy were synergistic with CI values in the range of 0.14 to 0.78. Moreover, apart from a few Dox concentrations combined with 700 nM Nic, all other combinations i.e., 38 combinatorial concentrations, were highly synergistic having CI < 0.5 (Figure 1J and Figure S1D). For SKBR3 cells, low concentrations of Dox (25–200 nM) combined with all the concentrations of Nic (300–800 nM) were synergistic with CI in the range of 0.58–0.98 (Figure 1K and Figure S1E). Further, for MCF7 cells, apart from a few concentrations of Dox combined with 600 nM Nic, all the combinatorial concentrations studied were synergistic with CI in the range of 0.42–0.97 (Figure 1L and Figure S1F). More specifically, in the case of MCF7 cells, of the 42 combinations studied, 37 were synergistic (Figure S1F). Taken together, these results imply that Nic, when sequentially combined with Dox (Nic → Dox), caused synergism, which led to enhanced cell cytotoxicity in cells of all the breast cancer types.

#### 3.1.2. Mode of Cell Death Caused by the Sequential Combination of Nic and Dox in Breast Cancer Cells

**Figure 2:**
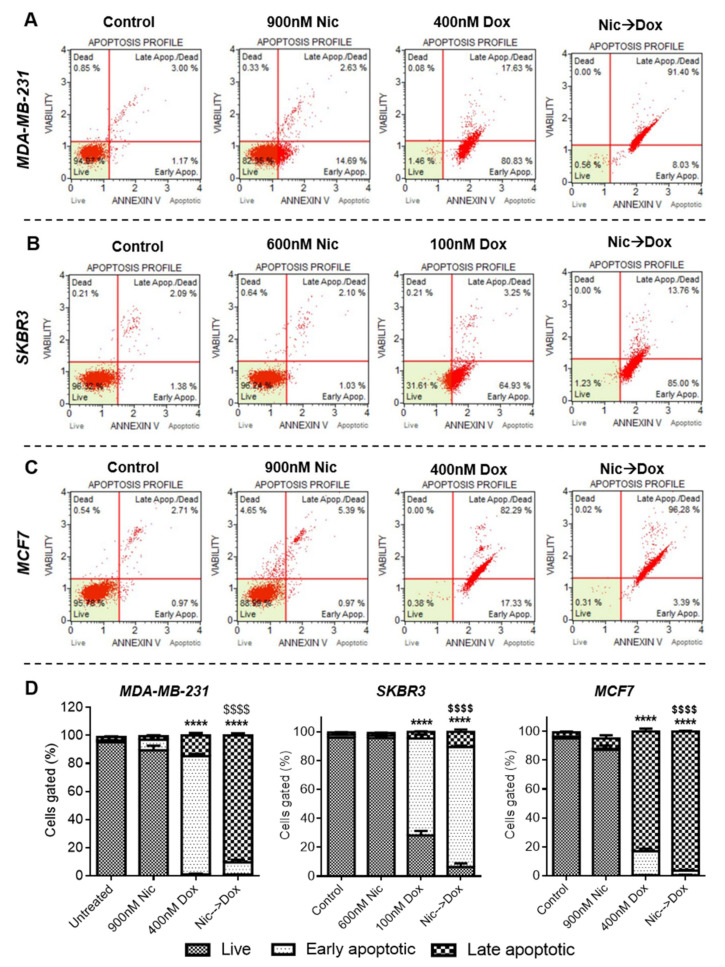
Apoptosis analysis of breast cancer cells after individual and sequential treatment of Nic and Dox at indicated doses for 24 h. Flow cytometer scatter plots of (**A**) MDA-MB-231, (**B**) SKBR3, and (**C**) MCF7 cells showing apoptosis profiles of different treatment groups. (**D**) Quantitative representation of apoptosis profiles shown in (**A**–**C**). **** indicates statistically significant increase (*p* < 0.0001) in percentage of total apoptotic cells in Dox and sequentially treated group with respect to Nic and untreated groups; $$$$ indicates statistically significant increase (*p* < 0.0001) in percentage of late apoptotic cells in sequentially treated group with respect to individual Dox group; (*n* = 3).

To decipher the mode of enhanced death of breast cancer cells in sequential combination of Nic and Dox, apoptosis analysis was performed. For this, each breast cancer cell line was treated with one synergistic pair of Nic and Dox with Nic concentrations being intermediate and Dox concentrations being on the lower side (MDA-MB-231: Nic-900 nM, Dox-400 nM; SKBR3: Nic-600 nM, Dox-100 nM; and MCF7: Nic-900 nM, Dox-400 nM). As demonstrated in the representative flow cytometer scatter plots of all the groups showing an apoptosis profile in MDA-MB-231 (Figure 2A), SKBR3 (Figure 2B), and MCF7 cells (Figure 2C), it was found that the apoptosis rates were significantly higher in the combination groups (Nic → Dox) compared to control, Nic, and Dox groups. Individual treatment of selected concentrations of Nic caused very minimal cell death of all the breast cancer cells studied (Figure 2). Dox treatment caused significant cell death of breast cancer cells; however, percentages of late apoptotic cells were significantly higher in combination treatment groups in all the cell lines (Figure 2D). More specifically, in MDA-MB-231 cells, Dox treatment had 98.46% of apoptotic cells, of which only 17.63% were in late apoptotic stage (Figure 2A). However, in the combination treatment group, 99.43% were apoptotic of which 91.4% of cells were in the late apoptotic stage (Figure 2A). Further, Dox treatment caused apoptosis of 68.18% SKBR3 cells, which increased to 98.76% in the combination treatment (Figure 2B). In MCF7 cells, individual treatment of Dox had 82.29% late apoptotic cells, whereas this percentage increased to 96.28% in the combination group (Figure 2C). Overall, these results imply that sequential combination of Nic and Dox significantly inhibited cell growth in all three subtypes of breast cancer mainly by inducing apoptosis (Figure 2D).

#### 3.1.3. Effect of Nic Treatment on Wnt/β-Catenin Signaling in Breast Cancer Cells

**Figure 3:**
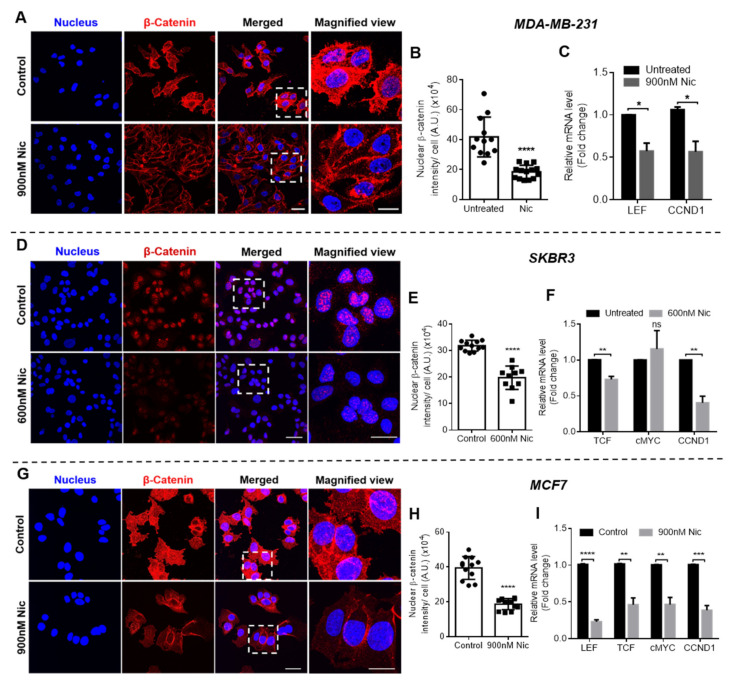
Wnt signaling analysis in breast cancer cells after 24 h treatment of Nic at indicated doses. Representative images of β-catenin immunostaining after 24 h treatment of Nic at selected doses in (**A**) MDA-MB-231, (**D**) SKBR3, and (**G**) MCF7 cells; scale bar—40 µm; magnified view—20 µm. Quantification of nuclear β-catenin from immunofluorescent images using MATLAB software in (**B**) MDA-MB-231, (**E**) SKBR3, and (**H**) MCF7 cells. Data represent mean values ± SD (n = 10–15 images). Gene expression analysis of Wnt/β-catenin signaling markers LEF, TCF, CCND1, and cMYC after 24 h treatment of Nic at indicated doses in (**C**) MDA-MB-231, (**F**) SKBR3, and (**I**) MCF7 cells. Normalized with respect to GAPDH expression. * (*p* < 0.05); ** (*p* < 0.01), *** (*p* < 0.001) and **** (*p* < 0.0001) indicate statistically significant difference with respect to untreated group; ns indicates no significant difference (*n* = 3).

Wnt signaling was investigated in all breast cancer cell types when treated with selected concentrations of Nic for 24 h to evaluate if the increased sensitivity of Nic-treated cells towards Dox is associated with Wnt signaling regulation. At the outset, immunostaining was performed to analyze β-catenin expression, which activates Wnt signaling when translocated to nucleus from cytoplasm. As shown in the confocal micrographs of β-catenin staining in MDA-MB-231 (Figure 3A), SKBR3 (Figure 3D), and MCF7 cells (Figure 3G), β-catenin expression reduced in Nic-treated cells when compared to untreated control in all the cell lines. Nuclear β-catenin intensity in the images was quantified using the MATLAB program and the results demonstrated that there was significant downregulation in the nuclear β-catenin intensity in Nic-treated groups in all the breast cancer cells when compared to control (Figure 3B,E,H). Further, gene expression analysis was performed for Wnt signaling markers LEF and TCF (transcription factors) and cMYC and CCND1 (cyclin D1), which are target genes of Wnt signaling. The results demonstrated that 24 h treatment of Nic caused significant downregulation of Wnt signaling markers in all the breast cancer cell types (Figure 3C,F,I). Specifically, LEF (0.57-fold) and CCND1 (0.56-fold) were downregulated in MDA-MB-231 cells (Figure 3C), TCF (0.72-fold) and CCND1 (0.40-fold) were downregulated in SKBR3 cells (Figure 3F), and all the markers LEF, TCF, cMYC, and CCND1 were downregulated 0.23, 0.46, 0.46, and 0.38-fold, respectively, in MCF7 cells (Figure 3I). TCF gene expression levels were undetectable in MDA-MB-231 and LEF was undetectable in SKBR3 cells. Further, there was no significant difference in cMYC levels in SKBR3 cells (Figure 3F).

Collectively, these results suggested that Nic treatment caused significant downregulation of Wnt/β-catenin signaling in all subtypes of breast cancer cells, which may have increased the sensitivity of breast cancer cells against Dox.

#### 3.1.4. Effect of Nic Treatment on Cell Cycle Progression in Breast Cancer Cells

**Figure 4:**
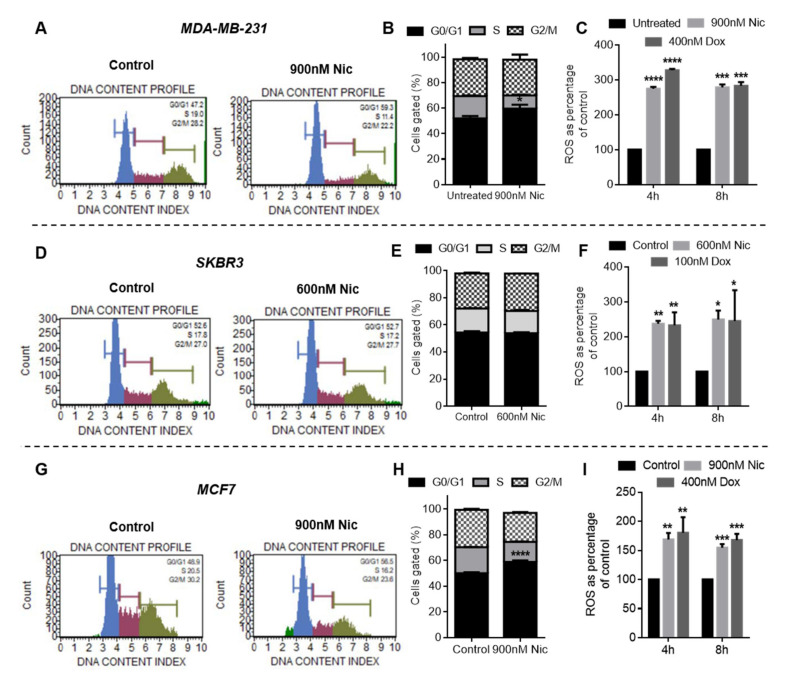
Cell cycle and ROS analysis in breast cancer cells. Cell cycle histograms of (**A**) MDA-MB-231, (**D**) SKBR3, and (**G**) MCF7 cells, when treated with selected concentrations of Nic for 24 h. Blue color indicates cells in G0/G1 phase, magenta color indicates cells in S phase, and green color indicates cells in G2/M phase. Quantitative representation of cell cycle histograms shown in (**A**,**D**,**G**) for (**B**) MDA-MB-231, (**E**) SKBR3, and (**H**) MCF7 cells. * (*p* < 0.05) and **** (*p* < 0.0001) indicate statistically significant increase in G0/G1 phase of the Nic-treated group with respect to the untreated group (n = 3). Reactive oxygen species (ROS) analysis of Nic- and Dox-treated cells at indicated doses for 4 h and 8 h through DCFDA assay in (**C**) MDA-MB-231, (**F**) SKBR3, and (**I**) MCF7 cells. * (*p* < 0.05); ** (*p* < 0.01), *** (*p* < 0.001), and **** (*p* < 0.0001) indicate statistically significant difference with respect to the untreated group (*n* = 3).

Wnt signaling analysis revealed that Nic treatment downregulated cyclin D1 in all the breast cancer cell types studied. Cyclin D1 is a major regulator of cell cycle progression that promotes G0/G1 to S phase transition in cell cycle [19]. Therefore, the effect of Nic treatment on cell cycle progression was investigated in breast cancer cells. Representative flow cytometer histograms and quantitative column plots of control and Nic-treated groups showing the DNA content profile indicated that the Nic treatment caused a significant increase in the percentage of cells arrested in the G0/G1 phase when compared to the untreated control in MDA-MB-231 (Figure 4A,B) and MCF7 cells (Figure 4G,H). In SKBR3 cells, though a significant percentage of cells was arrested in the G0/G1 phase in the Nic treatment group, there was no statistically significant difference between the control and Nic-treated groups (Figure 4D,E). These results imply that Nic treatment led to downregulation of CyclinD1, which in turn caused the arrest of breast cancer cells (MDA-MB-231 and MCF7) in the G0/G1 phase of their cell cycle.

#### 3.1.5. Reactive Oxygen Species (ROS) Generation in Nic- and Dox-Treated Breast Cancer Cells

Previous reports have shown that cells arrested in the G0/G1 phase undergo apoptosis under conditions that lead to DNA damage [20,21]. ROS is one such DNA damage condition known to be produced by anthracyclines such as doxorubicin [22]. Since cell viability and apoptosis analysis demonstrated that sequential combination of Nic and Dox (Nic → Dox) caused significantly enhanced death of breast cancer cells, we investigated whether the combinatorial efficacy of Nic and Dox is in part mediated by ROS generation. The results from the DCFDA assay (which majorly detects peroxide ions) suggested that both Nic and Dox generated a significantly enhanced amount of ROS in all breast cancer cell types after 4 and 8 h of treatment, which ranged 150–350% when compared to untreated control (Figure 4C,F,I). Moreover, DHE analysis (which majorly detects superoxide ions) also demonstrated similar results with both Nic and Dox generating significantly enhanced amount of ROS (180–540%) after 4 and 8 h of incubation in all the cell lines (Figure S2). Taken together, these results imply that consistently high amounts of ROS induced by Nic and Dox could have facilitated the process of apoptosis in all breast cancer cell types. Moreover, in the sequential treatment group (Nic → Dox), Nic treatment would lead to arrest of cells in the G0/G1 phase. These arrested cells, when exposed to ROS generated by Dox, would undergo apoptosis to eventually cause enhanced cell death.

Overall, these results suggested that sequential combination of Nic and Dox is efficacious at multiple combinatorial concentrations in all the three clinical subtypes of breast cancer. Sequential combination therapy induced apoptosis and caused synergistically enhanced death of cells of all the subtypes of breast cancer when compared to individual treatments. Further, delving into the mechanism of synergism between the two drugs, it was observed that the combinatorial efficacy was mediated through a similar mechanism in all the three subtypes, which involves downregulation of Wnt/β-catenin signaling, cell cycle arrest at G0/G1 phase by Nic treatment, a high amount of ROS production by Nic and Dox along with independent cytotoxicity of Dox.

### 3.2. Concurrent Treatment of Nic and Dox on Breast Cancer Cells

#### 3.2.1. Cytotoxicity Analysis of Individual Versus Concurrent Combination of Nic and Dox

**Figure 5:**
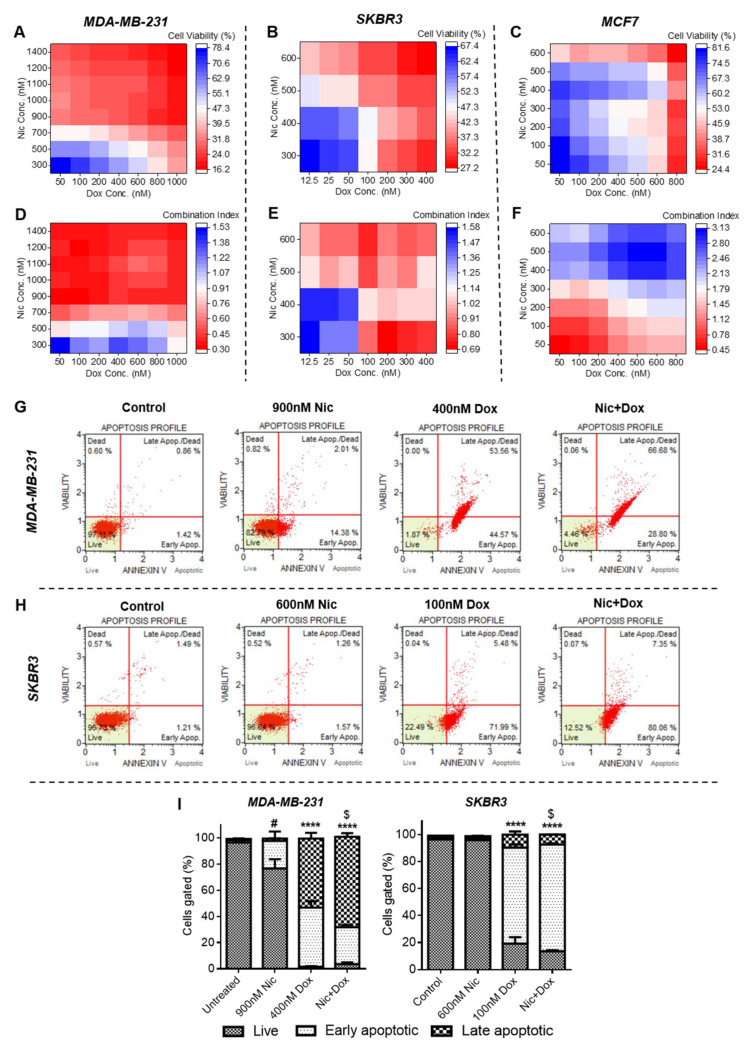
Effect of individual and concurrent treatment of Nic and Dox on breast cancer cells. Cell viability heat maps of concurrent treatment of Nic and Dox for 48 h in (**A**) MDA-MB-231, (**B**) SKBR3, and (**C**) MCF7 cells. Combination index heat maps of combination therapy in (**D**) MDA-MB-231, (**E**) SKBR3, and (**F**) MCF7 cells. Flow cytometer scatter plots of (**G**) MDA-MB-231 and (**H**) SKBR3 cells showing apoptosis profiles when treated with individual and concurrent treatment of Nic and Dox. (**I**) Quantitative representation of apoptosis profiles shown in G and H. **** (*p* < 0.0001) indicates statistically significant increase in percentage of apoptotic cells in Dox and combination groups with respect to Nic and untreated control; $ (*p* < 0.05) indicates statistically significant increase in percentage of late apoptotic cells in MDA-MB-231 and early apoptotic cells in SKBR3 cells with respect to Dox and # (*p* < 0.05) indicates statistically significant increase in percentage of apoptotic cells in the Nic group with respect to the untreated control (*n* = 3).

At the outset, individual cytotoxicity of Nic and Dox after 48 h of treatment (sequential treatment −24 h Nic → 24 h Dox = total 48 h) was evaluated for which breast cancer cells were incubated with different concentrations of Nic and Dox. As evident from the cell viability plots of Figure S3, Nic and Dox caused significant cell death at higher concentrations for all the breast cancer cell types. As shown in Figure S3, IC_50_ values of Nic were 1.24, 0.44, and 0.29 µM and of Dox were 0.64, 0.31, and 0.48 µM when treated against MDA-MB-231, SKBR3, and MCF7 cells, respectively. Subsequently, breast cancer cells were concurrently incubated with different concentrations of Nic and Dox for 48 h (Figure 5 and Figure S4). For MDA-MB-231 cells, in total, 56 concurrent combinatorial concentrations were used of which 47 combinations caused more than 50% cell death (Figure 5A and Figure S4A). More specifically, intermediate to higher concentrations of Nic (900–1400 nM) combined with all the concentrations of Dox (50–1000 nM) caused higher cytotoxicity (~≥70%) than lower concentrations of Nic combined with any concentration of Dox (Figure 5A and Figure S4A). Calculation of combination indices suggested that 47 combinations were synergistic with CI < 1 (0.29–0.95) of which 33 combinations were highly synergistic with CI < 0.5 (0.29–0.48) (Figure 5D and Figure S4D). Therefore, similar to sequential therapy, concurrent therapy of Nic and Dox was highly efficacious in MDA-MB-231 cells. The SKBR3 cells were treated with 28 concurrent combinations of Nic and Dox, of which 22 pairs caused greater than 50% cell death (Figure 5B and Figure S4B). More specifically, all the concentrations of Nic (300–600 nM) combined with intermediate to higher concentrations of Dox (100–400 nM) were more cytotoxic than low to intermediate concentrations of Nic combined with lower concentrations of Dox (Figure 5B and Figure S4B). Calculation of combination indices of all the combinatorial pairs revealed that out of 28 combinations tested, 15 were synergistic with CI < 1; however, none of the combinations were highly synergistic as CI values ranged between 0.5 and 1 (Figure 5E and Figure S4E). These results suggest that concurrent therapy was efficacious in SKBR3 cells; however, it was not as efficacious as it was in TNBC cells. Further, cytotoxicity analysis of 49 different combinatorial concentrations of Nic and Dox in MCF7 cells indicated that 16 combinatorial concentrations caused >50% cell death (Figure 5C and Figure S4C). However, higher concentrations of Dox combined with all concentrations of Nic were more efficacious (Figure 5C). Further, combination index plot demonstrated that of 49 combinations tested, 6 combinations were synergistic; however, cell death at these combinatorial concentrations was not very high and ranged from 19% to 36% (Figure 5F and Figure S4C,F). Therefore, these results imply that concurrent therapy was not efficacious in MCF7 cells.

Overall, these results indicate that concurrent combination of Nic and Dox significantly inhibited growth of all the breast cancer cell types. However, in the case of MDA-MB-231 and SKBR3 cells, the synergistic interaction between the two drugs led to high cell death, whereas while synergism was observed in few of the concentrations studied in MCF7 cells, it did not lead to high cell death.

#### 3.2.2. Mode of Cell Death Caused by Concurrent Combination of Nic and Dox in Breast Cancer Cells

Apoptosis analysis of the individual Nic, Dox, and selected concurrent combinatorial pairs (concentrations used were same as that used in sequential therapy) of Nic and Dox was performed in MDA-MB-231 and SKBR3 cells to decipher the mode of cell death in these cell lines. In the case of MCF7 cells, since the concurrent combinatorial pairs of Nic and Dox that showed synergism did not cause high cell death, apoptosis analysis was not performed. For MDA-MB-231 cells, the results demonstrated that 48 h of individual exposure of 900 nM Nic and 400 nM Dox caused 16.39% and 98.13% of apoptosis, respectively (Figure 5G). Of these, 2.01% and 53.56% of apoptotic cells were late apoptotic/dead in the Nic and Dox group, respectively (Figure 5G). Whereas, when concurrently treated with Nic and Dox, 95.48% cells were apoptotic of which 66.68% were late apoptotic/dead (Figure 5G). Moreover, apoptosis analysis of SKBR3 cells after 48 h treatment of individual and concurrent treatment of Nic and Dox suggested that Nic alone did not cause significant apoptosis (Figure 5H). Dox caused apoptosis of 77.47% cells, whereas combination of Nic and Dox caused apoptosis of 87.41% cells (Figure 5H). Statistical analysis suggested that the combinatorial group caused significantly enhanced apoptosis of MDA-MB-231 and SKBR3 cells when compared to both the individual treatments as well as untreated control (Figure 5I). Overall, these results indicated that concurrent combination of Nic and Dox caused significantly enhanced death of MDA-MB-231 and SKBR3 cells mainly by inducing apoptosis.

#### 3.2.3. Effect of Nic Treatment Either Alone or in Combination with Dox on Wnt/β-Catenin Signaling in Breast Cancer Cells

**Figure 6:**
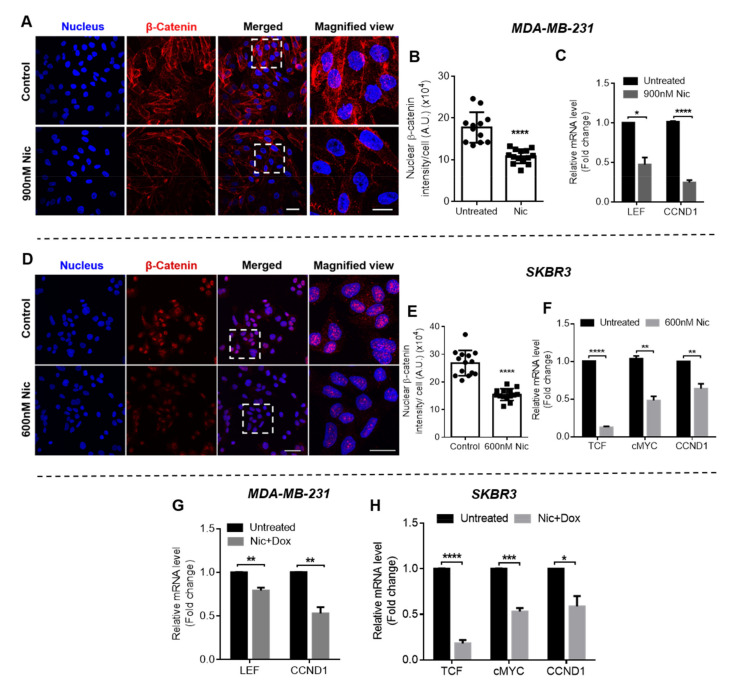
Wnt signaling analysis in breast cancer cells after individual and concurrent treatment of Nic and Dox. Representative images of β-catenin immunostaining of (**A**) MDA-MB-231 and (**D**) SKBR3 cells after 48 h treatment of Nic (scale bar- 40 µm; magnified view- 20 µm). Quantification of nuclear β-catenin from immunofluorescent images shown in (**A**,**D**) using MATLAB software in (**B**) MDA-MB-231 and (**E**) SKBR3 cells. Data represents mean values ± SD (*n* = 12–14 images). Gene expression analysis of Wnt/β-catenin signaling markers LEF, TCF, cMYC, and CCND1 after 48 h treatment with (**C**,**F**) Nic; and (**G**,**H**) combination of Nic and Dox, in MDA-MB-231 and SKBR3 cells. Normalization performed with GAPDH expression. * (*p* < 0.05); ** (*p* < 0.01), *** (*p* < 0.001), and **** (*p* < 0.0001) indicate statistically significant difference with respect to the untreated group (*n* = 3).

In the case of concurrent therapy, mechanism of synergism was evaluated only for MDA-MB-231 and SKBR3 cells. Since, Nic and Dox did not show effective synergism in MCF7 cells, subsequent experiments related to the mechanistic understanding of the synergism was not performed in this case. Confocal micrographs of MDA-MB-231 and SKBR3 cells showing β-catenin immunostaining suggested that Nic reduced nuclear β-catenin expression after 48 h of treatment when compared to untreated control (Figure 6A,D). Nuclear β-catenin intensity quantified from these images suggested that Nic treatment significantly reduced nuclear β-catenin when compared to untreated control in both the cell lines (Figure 6B,E). Further, gene expression analysis of Wnt markers suggested that Nic significantly downregulated LEF (0.47-fold) and CCND1 (0.24-fold) in MDA-MB-231 cells when incubated for 48 h (Figure 6C). Moreover, the efficacy of Nic in downregulating Wnt signaling was also evaluated when used along with Dox. The results demonstrated that 900 nM Nic significantly downregulated LEF and CCND1 to 0.79 and 0.52-fold, respectively, when used in combination with Dox for 48 h (Figure 6G). Further, in SKBR3 cells, Nic caused significant downregulation of TCF, cMYC, and CCND1 after 48 h of treatment both alone (Figure 6F) as well as when used in combination with Dox (Figure 6H). Fold change in TCF, cMYC, and CCND1 levels were 0.12, 0.48, and 0.63, respectively, when treated with Nic and were 0.18, 0.53, and 0.58, respectively, when treated with Nic+ Dox. TCF and LEF levels were undetectable in MDA-MB-231 and SKBR3 cells respectively.

Collectively, these results imply that 48 h treatment of Nic significantly downregulated Wnt signaling in MDA-MB-231 and SKBR3 cells. Moreover, the results depicted in Figure 6G,H confirmed that Nic caused significant downregulation of Wnt signaling in the presence of Dox as well.

#### 3.2.4. Effect of Nic Treatment Either Alone or in Combination with Dox on Cell Cycle Progression in Breast Cancer Cells

**Figure 7:**
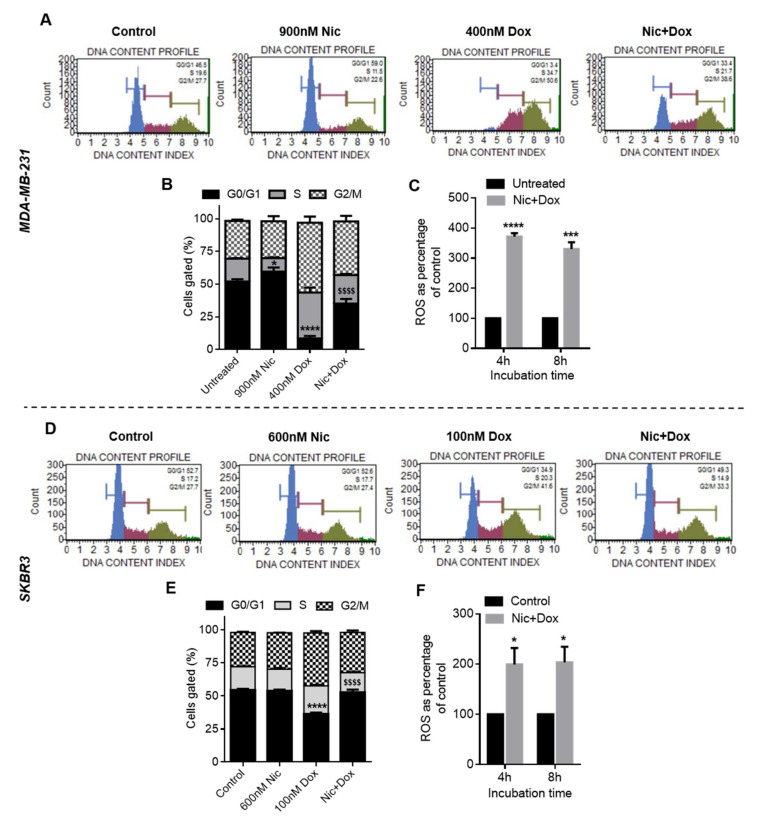
Cell cycle and ROS analysis in breast cancer cells after individual and concurrent treatment of Nic and Dox. Cell cycle histograms of (**A**) MDA-MB-231 and (**D**) SKBR3 cells, when treated with selected concentrations of Nic, Dox, and concurrent combination of Nic and Dox (Nic + Dox) for 24 h. Quantitative representation of cell cycle analysis for (**B**) MDA-MB-231 and (**E**) SKBR3. * (*p* < 0.05) indicates statistically significant increase in G0/G1 phase of the Nic-treated group with respect to the untreated group; **** (*p* < 0.0001) indicates a statistically significant decrease in the G0/G1 phase of the Dox-treated group with respect to all other groups and $$$$ (*p* < 0.0001) indicates a statistically significant increase in the G0/G1 phase of the Nic + Dox-treated group with respect to the Dox group (*n* = 3). Reactive oxygen species (ROS) analysis of Nic+Dox-treated cells at indicated doses for 4 h and 8 h through DCFDA assay in (**C**) MDA-MB-231, and (**F**) SKBR3 cells. * (*p* < 0.05); *** (*p* < 0.001), and **** (*p* < 0.0001) indicate a statistically significant difference with respect to the untreated group (*n* = 3).

Cell cycle progression of MDA-MB-231 and SKBR3 cells were evaluated after individual as well as concurrent treatment of Nic and Dox. The results demonstrated that in MDA-MB-231 cells, Nic treatment caused 59% cells to be arrested in G0/G1 phase, whereas the Dox group had significantly low i.e., 3.4% of cells in G0/G1 phase (Figure 7A,B). Further, there was a significant increase in the percentage of G0/G1 cells (33.4%) in concurrent combination group when compared to Dox. Moreover, there was a significant decrease in the percentage of cells in the S and G2/M phase in the concurrent treatment group when compared to Dox (Figure 7A,B). Further, in SKBR3 cells, representative flow cytometer histograms suggested that percentage of cells in G0/G1 phase decreased in the Dox treatment group when compared to Nic and control (Figure 7D). Further, G0/G1 arrested cells increased in the combination group when compared to Dox. As demonstrated in Figure 7E, the percentage of cells in the G0/G1 phase in Dox group (i.e., 34.9%) was significantly reduced when compared to Nic and control (52.6%). Moreover, the increase in the number of G0/G1 cells in the combination group (i.e., 49.3%) was significant when compared to the Dox group. Overall, these results suggested that the increase in the percentage of cells arrested in the G0/G1 phase in combination groups is attributable to Nic, as the cell cycle arrest at the G0/G1 phase was very low when treated with Dox individually.

#### 3.2.5. ROS Generation by Concurrent Treatment of Nic and Dox in Breast Cancer Cells

Since cells were exposed to both the drugs simultaneously in concurrent therapy, ROS analysis was performed when MDA-MB-231 and SKBR3 cells were co-incubated with Nic and Dox. DCFDA and DHE results demonstrated that combinatorial concentrations generated significantly higher amount of ROS after 4 h of treatment, which was consistent for 8 h of investigation in both the cell lines (Figure 7C,F and Figure S5). Specifically, in MDA-MB-231 cells, the levels of ROS ranged from 288–370% and 330–414% after 4 and 8 h of Nic+Dox incubation (Figure 7C and Figure S5A). Further, in SKBR3 cells, the levels ranged 199–368% and 203–257% after 4 and 8 h of incubation, respectively (Figure 7F and Figure S5B).

Collectively, these results confirmed that the concurrent presence of Nic and Dox did not hamper each other’s efficacy. Rather, concurrent combination of Nic and Dox induced apoptosis and caused synergistically enhanced death of MDA-MB-231 and SKBR3 cells at multiple combinatorial concentrations. Further, mechanistic understanding revealed that the combination of drugs showed a similar mechanism of action irrespective of their treatment regimen (sequential or concurrent) and time of incubation (24 h or 48 h).

## 4. Discussion

The complexity of the cancer genome, the heterogeneity of the cancer cell and tumor microenvironment, and cancer drug resistance have made single agent therapies inefficient [23]. As a consequence, there is an unmet need to develop an efficient combination therapy, with clinically relevant targets and associated targeting agents. In this regard, cell signaling pathways have attracted much attention as the aberrantly expressed signaling pathways act as major cancer drivers. They not only contribute to the development of resistance against the given therapy but are also responsible for the molecular heterogeneity in cancer population and the oncogenic signature of cancer cells [4]. Wnt/β-catenin is one such aberrantly expressed signaling pathway in breast cancer, which is found to be associated with enhanced tumorigenesis, invasion, and chemoresistance of cancer cells [24]. Therefore, a combinatorial approach of targeting Wnt signaling combined with a conventional anticancer agent might improve the therapeutic outcome. Nic has shown promising Wnt signaling inhibition in many pre-clinical studies in multiple cancer types [25]. Hence, in the present study, we developed a combination therapy based on Nic and Dox, which has hitherto not been explored for cancer therapy. Although Dox is the standard of care for breast cancer in the clinic [26], high dosage and non-specific biodistribution of Dox can cause severe life-threatening side effects such as cardiotoxicity [27,28]. Therefore, it is desirable to reduce the concentration of Dox required for effective therapy. To this end, we used Nic to target Wnt signaling, thereby enhancing the possibility of improving the therapeutic efficacy of Dox at lower concentrations while obtaining the added advantage of downregulating Wnt signaling.

Cytotoxicity analysis suggested that individual Nic and Dox caused significant cytotoxicity to breast cancer cells. However, different clinical subtypes of breast cancer cells responded with differential sensitivity towards Nic and Dox. Of all the three subtypes, MDA-MB-231 cells exhibited considerably less sensitivity towards both the drugs and had the highest IC_50_ values, SKBR3 cells were relatively more sensitive and had the lowest IC_50_ values, whereas MCF7 cells had intermediate sensitivity of the three clinical subtypes except for 48 h IC_50_ of Nic, which was the lowest for MCF7 cells. Later, cytotoxicity analysis of the combinatorial regimens demonstrated that both sequential (Nic → Dox) and concurrent (Nic + Dox) combinations of Nic and Dox were more potent in inhibiting growth of breast cancer cells at concentrations much lesser than their respective IC_50_ values. It is known that two drugs are synergistic if CI < 1, and the lower the CI is, the better is the synergism between the two drugs [18]. Based on the calculation of combination indices, multiple combinations were synergistic in both the treatment regimens in MDA-MB-231 and SKBR3 cells. In MCF7 cells, though both sequential and concurrent combinatorial concentrations caused significant cell death, the combinations were synergistic only in sequential therapy, as in the concurrent regimen, cytotoxic combinatorial pairs were associated with CI values greater than 1. Therefore, the extent of synergism varied between subtypes. Interestingly, TNBC cell line MDA-MB-231, which had the least sensitivity towards individual drugs, demonstrated the highest sensitivity and synergism against both the combinatorial treatment regimens among all subtypes. This is exciting, as TNBC, which lacks any targeted therapy, is the breast cancer subtype most difficult to treat in the clinic. On the other hand, the HER2-positive breast cancer cell line, SKBR3, which demonstrated the highest sensitivity towards individual drugs at 24 h, had the lowest sensitivity and synergism in the sequential treatment regimen. Further, the HR-positive cell line, MCF7, showed better sensitivity towards sequential combination when compared to SKBR3 cells; however, despite having the highest sensitivity towards Nic at 48 h, it did not show efficacious synergism towards concurrent treatment. Collectively, we observed a subtype-dependent efficacy of the combination of Nic and Dox, which could be attributed to the molecular differences in these clinical subtypes [29]. Notably, within a subtype, treatment schedule, incubation time, and concentrations are important factors, as variations in them led to changes in synergism.

Further, conventional methods to analyze combinatorial efficacy most often involve calculation of the combination index for 50% cell death or make use of fixed combinatorial ratios [30,31,32,33]. However, we screened many combinatorial concentrations of Nic and Dox in large number of ratios and calculated combination indices for all the combinations tested. This ensures the study is more comprehensive as it has a greater coverage of concentrations and ratios. Further, the study also highlights the importance of regulating the concentrations and ratios of Nic and Dox to obtain synergism as few concentrations and ratios led to additive or antagonistic effects. However, the non-synergistic concentrations and ratios were not further pursued due to their lack of therapeutic potential. Therefore, this study demonstrated the importance of comprehensive analysis to enable the choice of an appropriate treatment schedule, incubation time, as well as concentrations of Nic and Dox to be used for effective combination therapy.

Subsequently, to decipher the mechanism of synergism between Nic and Dox, one synergistic pair of Nic and Dox (with intermediate concentrations of Nic and low concentrations of Dox) was selected for each subtype of breast cancer. First, the mode of cytotoxicity of cancer cells was investigated and the results demonstrated that the enhanced cytotoxicity of Nic and Dox combination in both the sequential as well as the concurrent regimen was indeed mediated by apoptosis. Based on the cell viability and apoptosis studies, it can be inferred that Nic treatment sensitized breast cancer cells towards Dox, and as a consequence the combination irrespective of regimen (Nic → Dox and Nic + Dox), led to enhanced cytotoxicity mediated by apoptosis. Further, since downregulation of Wnt signaling is expected to increase the sensitivity of Dox for cancer cells [34], Wnt signaling was investigated after Nic treatment. For sequential therapy (Nic → Dox), cancer cells were incubated with Nic for 24 h, and for concurrent therapy (Nic + Dox), the cells were exposed to Nic for 48 h. The results obtained for Wnt signaling markers LEF, TCF, cMYC, and CCND1 through RT-PCR and β-catenin through immunostaining suggested that Nic efficiently downregulated Wnt signaling in breast cancer cells in both regimens with a greater downregulation observed with the increase in duration of exposure. Since the sequential regimen involves pre-exposure of Nic to sensitize cells towards Dox, the possibility of Dox interfering with Nic does not exist. However, in the concurrent regimen, cells are simultaneously exposed to Nic and Dox, thereby leading to the possibility of Dox interference with Nic activity. Therefore, Wnt signaling was also investigated in MDA-MB-231 and SKBR3 cells after co-incubation with both the agents to understand if Dox interferes with Nic activity in the Nic+Dox regimen. The RT-PCR results revealed that Nic significantly downregulated Wnt signaling in the presence of Dox as well. This proves that Dox does not adversely affect the activity of Nic, which is important for the concurrent combination regimen to be effective. Further, RT-PCR data revealed that among other Wnt target genes, Nic also downregulated CyclinD1 (CCND1), which promotes the G0/G1 to S phase transition in the cell cycle. Therefore, cell cycle phase distribution was assessed after individual Nic treatment (as in sequential Nic → Dox therapy, cells were first incubated with Nic only for 24 h) as well as concurrent (Nic + Dox) treatment. It was observed that there was a significant increase in the percentage of cells arrested in the G0/G1 phase after Nic treatment for 24 h when compared to the control in MDA-MB-231 and MCF7 cells. In the case of SKBR3 cells, Nic treatment caused significant downregulation of Wnt signaling including downregulation of CyclinD1. However, there was no significant difference in the percentage of cells arrested in the G0/G1 phase in the Nic-treated group when compared to the untreated group. This could be because of other parallel signaling pathways working in cancer cells and needs further investigation. This implies that the downregulation of CyclinD1 resulted in cell cycle arrest of breast cancer cells in the G0/G1 phase of their cell cycle. Moreover, individual Dox treatment led to a significant decrease in the percentage of cells in the G0/G1 when compared to control and Nic groups and significant increase in G2/M phase cells with respect to control, Nic, and Nic + Dox groups. Previous studies have also demonstrated that Nic causes arrest of cells in the G0/G1 phase of cell cycle [35,36] and Dox causes arrest of cells in the G2/M phase of cell cycle in mammalian cells [37,38]. However, Nic + Dox groups caused a significant increase in the number of cells in the G0/G1 phase when compared to the Dox group, though the number was lower compared to the Nic-only group. This implies that the presence of Nic in the concurrent therapy (Nic + Dox) caused a significant increase in the number of G0/G1 cells when compared to the Dox group, confirming that the downregulation of CyclinD1 led to cell cycle arrest in Nic + Dox treatment as well, which eventually facilitated the effectiveness of this regimen.

It is known that cell cycle checkpoints repair damaged DNA and that cells enter the apoptotic pathway if damaged DNA is not repaired properly [39,40]. Studies suggest that cells at the G1 checkpoint have reduced capability to repair DNA double strand breaks (DSBs) when compared to the G2 checkpoint [41]. This is so because the G1 checkpoint only has a single copy of each chromatid and therefore non-homologous DNA end joining (NHEJ) is the primary repair pathway of DSBs at the G1 checkpoint [42]. Since, NHEJ is error-prone, DNA repair is less efficient at the G1 checkpoint [43]. On the other hand, the G2 checkpoint has two sister chromatids, hence one sister chromatid can participate in the repair of another damaged chromatid. Therefore, at the G2 checkpoint, DSBS are primarily repaired by the homologous recombination (HR) pathway, which is an error-free pathway [44]. Therefore, collectively, DNA repair at the G1 checkpoint of cell cycle progression is less efficient and can be faulty when compared to the G2 checkpoint. This suggests that cells arrested in the G0/G1 phase are more prone to apoptosis if they encounter a DNA-damaging condition, which generates DSBs.

The generation of reactive oxygen species (ROS) is one such DNA damaging condition that generates DSBs [43,45]. Previous studies have reported ROS generation by Dox as well as Nic [22,46]. Since breast cancer cells treated with a combination of Nic and Dox (irrespective of regimen) had a significantly higher number of cells in the G0/G1 phase, the contribution of ROS to the significantly enhanced apoptosis of cancer cells was investigated. The results demonstrated that both Nic and Dox generated a significantly higher amount of ROS, which was at least double the amount present in the untreated control group over 8 h of investigation. Previous studies have demonstrated that such high levels of ROS are toxic to cells [47]. Therefore, the results suggested that ROS generated by Nic and Dox may be one of the important factors that caused the enhanced death of breast cancer cells in both the combination regimens.

**Figure 8:**
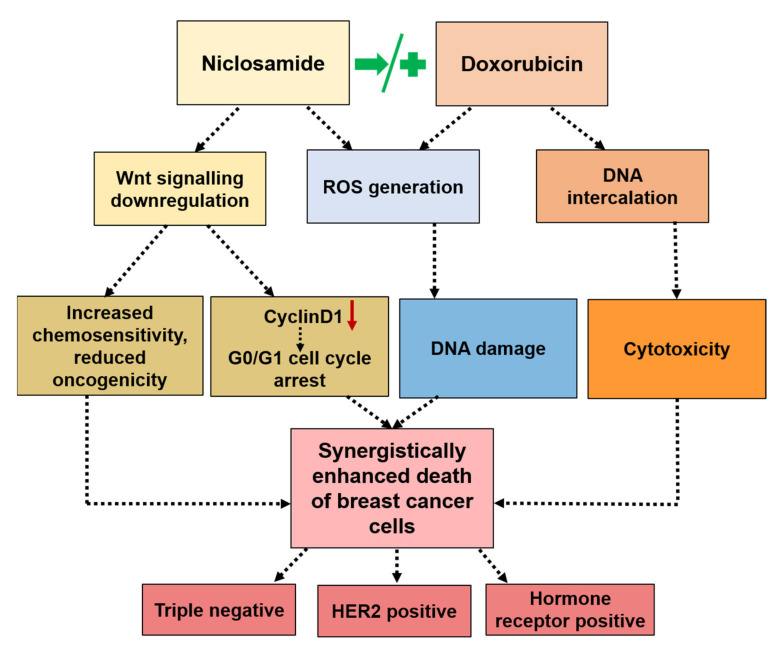
Mechanism of synergism between Nic and Dox in both sequential (Nic → Dox) and concurrent (Nic+Dox) treatment regimens.

Taken together, the results suggested the following mechanism of synergism in combination regimens. In sequential therapy (Nic → Dox), Nic caused significant downregulation of Wnt signaling, which made cancer cells chemo sensitive and less oncogenic. Further, Nic treatment caused cell cycle arrest at G0/G1 phase, which made these cells more prone to apoptosis in the event of a DNA damage condition. Incubating such chemo-sensitive and G0/G1-arrested cells with Dox led to significantly higher apoptosis due to enhanced inherent cytotoxicity of Dox as well as the generation of a high amount of ROS (Figure 8). In concurrent therapy (Nic + Dox), Nic caused a greater downregulation of Wnt signaling and consequential cell cycle arrest at the G0/G1 phase. The concurrent presence of Dox caused enhanced death of chemo-sensitive cancer cells as well as G0/G1 arrested cells through generation of high amount of ROS (Figure 8). Further, it is highly improbable that all the cancer cells in a tumor microenvironment will have a similar and synchronized response to the administered treatment as it is well known that the cancer cell population is highly heterogenous in terms of their responses to therapies as well as their oncogenic properties. Therefore, it can be expected that sequential therapy may cause slower tumor reduction and reduced off-target toxicity, whereas concurrent presence of Nic and Dox may cause faster tumor reduction; however, off-target toxicity load will also be higher in this case. Taken together, the choice of treatment regimen would depend on the patient and tumor characteristics.

Moreover, this study has demonstrated the effectiveness of the combination of Nic and Dox in both treatment regimens for breast cancer. However, the combination has the potential to be explored for multiple cancer types that are characterized by dysregulated Wnt signaling such as colorectal cancer, prostate cancer, lung cancer, ovarian cancer, leukemia, and glioblastoma [11,48].

## 5. Conclusions

We have developed a versatile combination therapy based on Nic and Dox, which showed a synergistic effect against all the three clinical subtypes of breast cancer at multiple combinatorial concentrations and both (sequential and concurrent) treatment regimens. Further, we elucidated the common mechanism of synergism in all the subtypes in both the regimens, which involves downregulation of Wnt signaling and cell cycle arrest at the G0/G1 phase by Nic, generation of a high amount of ROS by both Nic and Dox, and inherent cytotoxicity of Dox, which collectively induced significantly enhanced apoptosis of breast cancer cells. Overall, the results demonstrated that the combination of Nic and Dox holds potential to be explored further in preclinical studies for its development as a treatment option in breast cancer therapy.

## Data Availability

The data presented in this study are available in “A synergistic combination of niclosamide and doxorubicin as an efficacious therapy for all clinical subtypes of breast cancer” or in the Supplementary Materials of the same article.

## Supplementary Materials

The following are available online at https://www.mdpi.com/article/10.3390/cancers13133299/s1, Figure S1: Effect of sequential treatment of Nic and Dox on breast cancer cells, Figure S2: ROS analysis in breast cancer cells, Figure S3: Effect of individual treatment of Nic and Dox on breast cancer cells for 48 h, Figure S4: Effect of concurrent treatment of Nic and Dox on breast cancer cells, Figure S5: ROS analysis in breast cancer cells when concurrently treated with Nic and Dox, Table S1: List of primer sequences.
