# Supplementary material for: A Synergistic Combination of Niclosamide and Doxorubicin as an Efficacious Therapy for All Clinical Subtypes of Breast Cancer
Source: Cancers (Basel). 2021 Jun 30;13(13):3299. doi: 10.3390/cancers13133299 (PMC8268129; doi:10.3390/cancers13133299)
Supplement: Supplementary file 1 [file cancers-13-03299-s001.zip › cancers-1248363-supplementary.pdf]

Supplementary Material

A synergistic combination of niclosamide and doxorubicin as an efficacious therapy for all clinical subtypes of breast cancer

*Garima Lohiya and Dhirendra S. Katti**

Supplementary figures

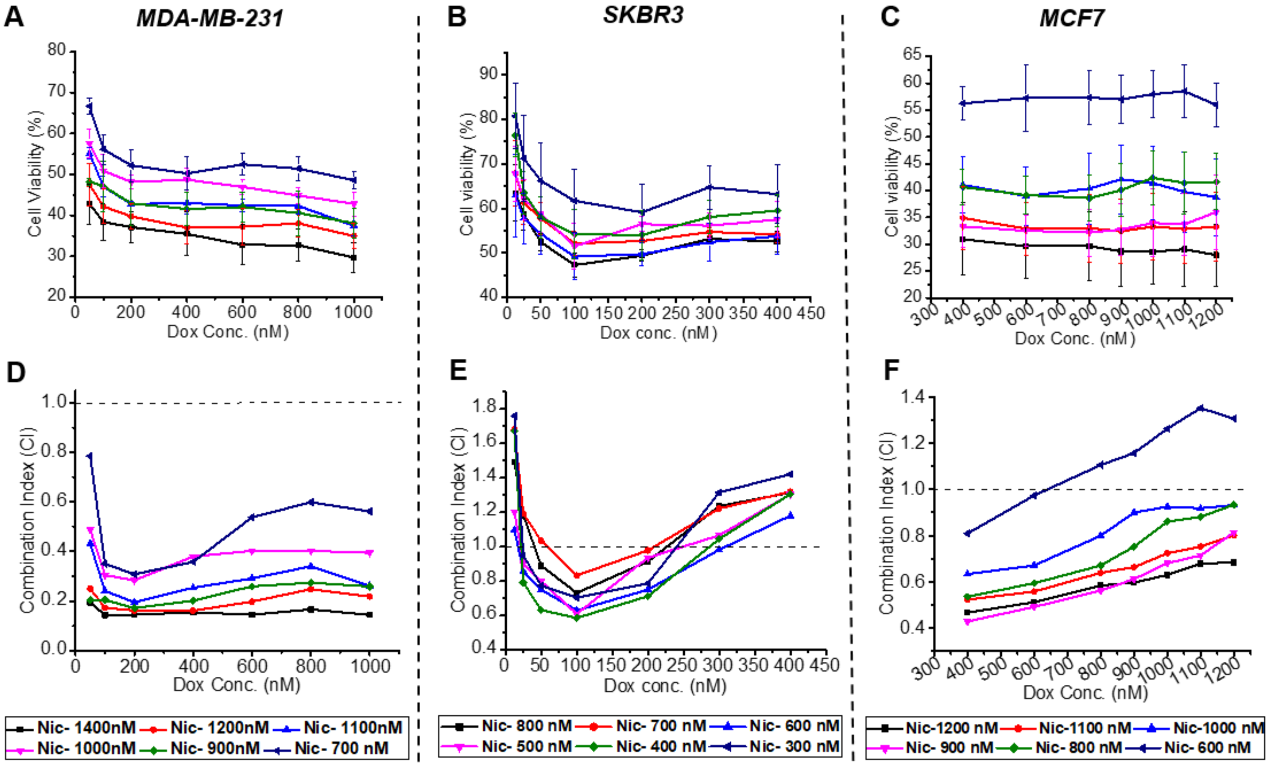

Figure S1. Effect of sequential treatment of Nic and Dox on breast cancer cells.

Cell viability line plots of different sequential combinatorial concentrations of Nic and Dox (24 h of Nic treatment followed by 24 h of Dox treatment) treated against A) MDA-MB-231, B) SKBR3 and C) MCF7 cells. Combination index line plots for D) MDA-MB-231, E) SKBR3 and F) MCF7 cells.

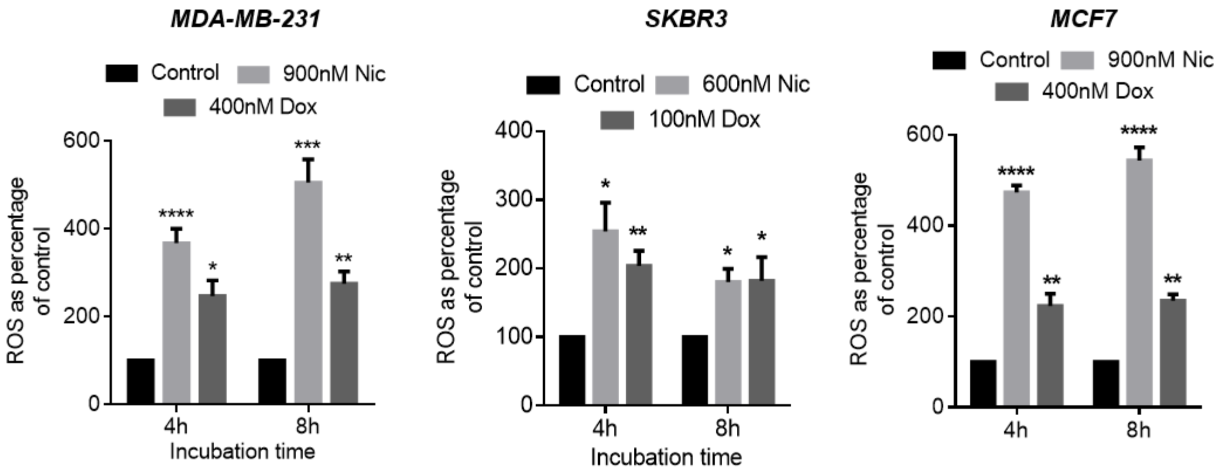

Figure S2. ROS analysis in breast cancer cells.

ROS analysis of Nic and Dox treated cells at indicated doses for 4 h and 8 h through DHE assay in MDA-MB-231, SKBR3 and MCF7 cells. *(p<0.05); **(p<0.01), ***(p<0.001) and ****(p<0.0001) indicate statistically significant difference with respect to untreated control group (n=3).

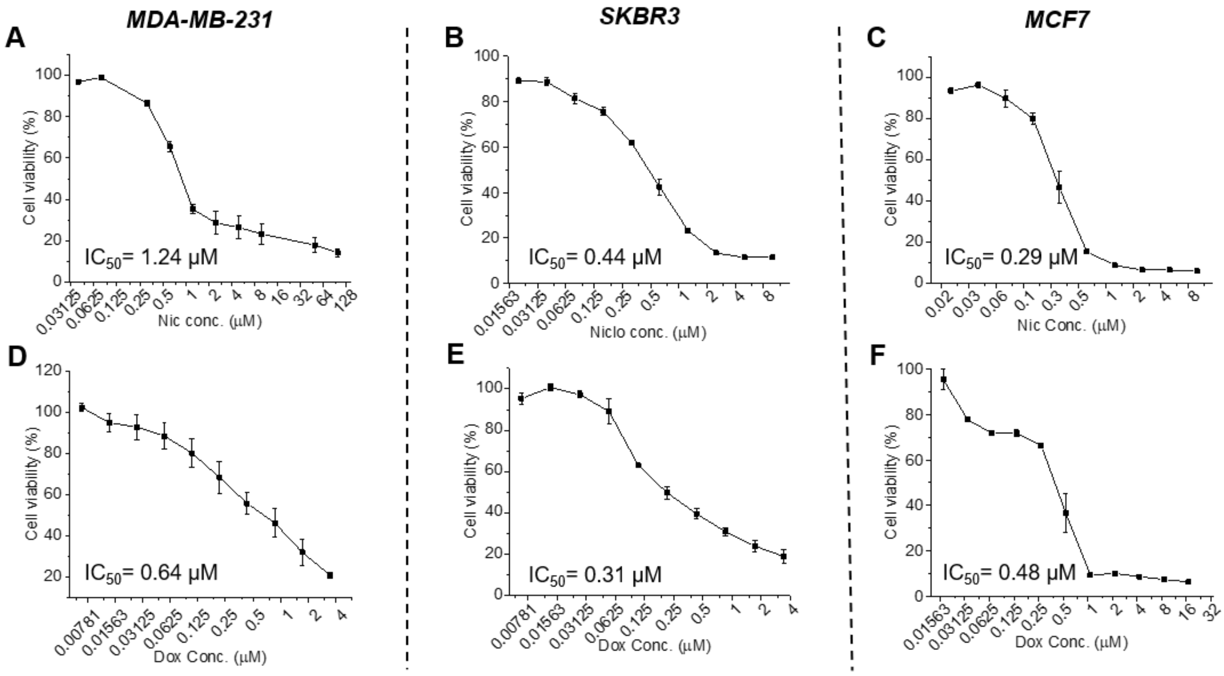

Figure S3. Effect of individual treatment of Nic and Dox on breast cancer cells for 48 h.

In vitro cytotoxicity of individual A-C) Nic and D-F) Dox on MDA-MB-231, SKBR3 and MCF7 cells at different doses for 48 h (n=3).

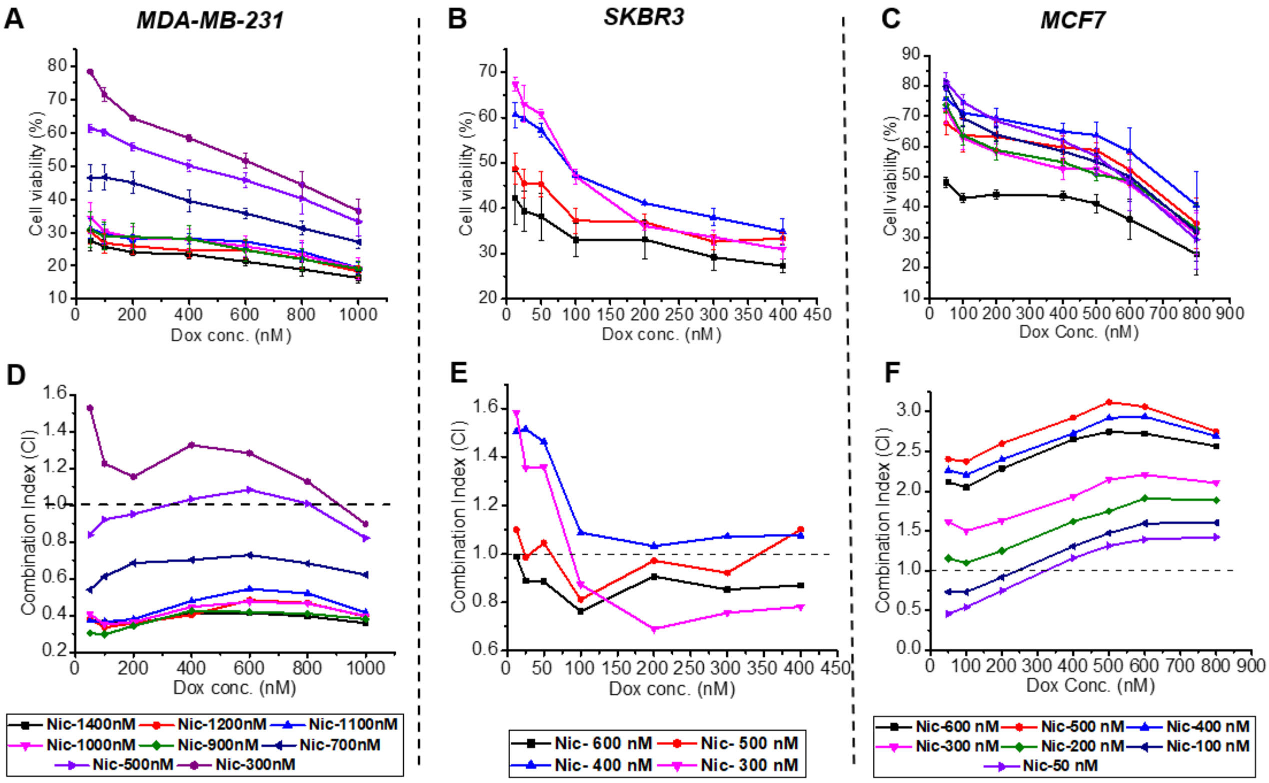

Figure S4. Effect of concurrent treatment of Nic and Dox on breast cancer cells.

Cell viability line plots of different concurrent combinatorial concentrations of Nic and Dox for 48 h treated against A) MDA-MB-231, B) SKBR3 and C) MCF7 cells. Combination index line plots of combination therapy for D) MDA-MB-231, E) SKBR3 and F) MCF7 cells.

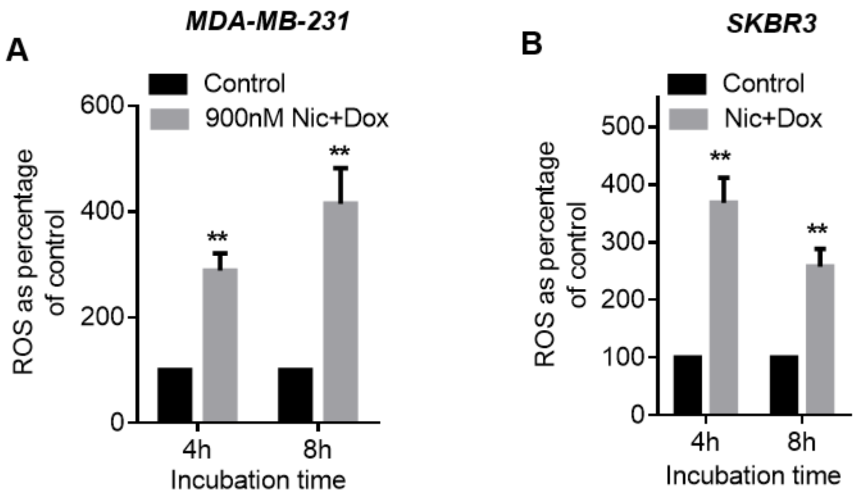

Figure S5. ROS analysis in breast cancer cells when concurrently treated with Nic and Dox.

Reactive oxygen species (ROS) analysis of Nic+Dox treated cells at indicated doses for 4 h and 8 h through DHE assay in A) MDA-MB-231, and B) SKBR3 cells. ** indicates statistically significant difference ($p<0.01$) with respect to untreated control group (n=3).

Table S1. List of primer sequences.

Name	Sequence
**Lymphoid enhancer factor (LEF)**	F: 5' AATGAGAGCGAATGTCGTTGC 3'
	R: 5' GCTGTCTTTCTTTCCGTGCTA 3'
**T-cell factor (TCF)**	F: 5' CTGCCTTAGGGACGGACAAAG 3'
	R: 5' TGCCAAAGAAGTTGGTCCATTTT 3'
**cellular Myelocytomatosis (c-Myc)**	F: 5' CATACATCCTGTCCGTCCAAG 3'
	R: 5' GAGTTCCGTAGCTGTTCAAGT 3'
**Axix inhibition protein2 (Axin2)**	F: 5' GAGTGGACTTGTGCCGACTTCA 3'
	R: 5' GGTGGCTGGTGCAAAGACATAG 3'
**CyclinD1 (CCND1)**	F: 5' GCTGCGAAGTGGAAACCATC 3'
	R: 5' CCTCCTTCTGCACACATTTGAA 3'
